# Cost of Acute and Sequelae Care for Japanese Encephalitis Patients, Bangladesh, 2011–2021

**DOI:** 10.3201/eid2912.230594

**Published:** 2023-12

**Authors:** Rebeca Sultana, Rose Slavkovsky, Md. Redowan Ullah, Zareen Tasnim, Sharmin Sultana, Shifat Khan, Tahmina Shirin, Shamsul Haque, Md. Tanvir Hossen, Md. Monjurul Islam, Jesmin Ara Khanom, Abrarul Haque, Arifa Nazneen, Nadia Ali Rimi, Kamal Hossain, Md. Tanbirul Islam, Shariful Hasan, Md. Shameem Yazdany, Md. Shamim Ahsan, Kamran Mehedi, Anthony A. Marfin, G. William Letson, Clint Pecenka, An Le Thanh Nguyen

**Affiliations:** icddr,b, Dhaka, Bangladesh (R. Sultana, M.R. Ullah, Z. Tasnim, S. Khan, A. Haque, A. Nazneen, N.A. Rimi, K. Hossain);; PATH, Seattle, Washington, USA (R. Slavkovsky, A.A. Marfin, G.W. Letson, C. Pecenka);; Institute of Epidemiology Diseases Control and Research, Dhaka (S. Sultana, T. Shirin);; Maternal, Newborn, Child and Adolescent Health, Dhaka (M.S. Haque, M.T. Hossen, M.M. Islam, J.A. Khanom);; World Health Organization, Dhaka (M.T. Islam);; Rangpur Medical College Hospital, Rangpur, Bangladesh (S. Hasan);; Rajshahi Medical College Hospital, Rajshahi, Bangladesh (M.S. Yazdany);; Chattogram Medical College Hospital, Chittagong, Bangladesh (M.S. Ahsan);; PATH, Dhaka (K. Mehedi);; PATH, Ho Chi Minh City, Vietnam (A.L.T. Nguyen)

**Keywords:** Japanese encephalitis, encephalitis virus, Japanese, cost of illness, Japanese encephalitis vaccines, viruses, Bangladesh

## Abstract

Japanese encephalitis (JE) is associated with an immense social and economic burden. Published cost-of-illness data come primarily from decades-old studies. To determine the cost of care for patients with acute JE and initial and long-term sequelae from the societal perspective, we recruited patients with laboratory-confirmed JE from the past 10 years of JE surveillance in Bangladesh and categorized them as acute care, initial sequalae, and long-term sequelae patients. Among 157 patients, we categorized 55 as acute, 65 as initial sequelae (53 as both categories), and 90 as long-term sequelae. The average (median) societal cost of an acute JE episode was US $929 ($909), of initial sequelae US $75 ($33), and of long-term sequelae US $47 ($14). Most families perceived the effect of JE on their well-being to be extreme and had sustained debt for JE expenses. Our data about the high cost of JE can be used by decision makers in Bangladesh.

Japanese encephalitis (JE) is among the most common viral causes of encephalitis in the world; an estimated 67,900 new cases of JE occur annually in JE-endemic countries, and case-fatality rates are 20%–30% (*1*,*2*). Among JE survivors, 30%–50% experience long-term neuropsychological sequelae (*1*,*3*,*4*). Clinical signs/symptoms develop in only ≈1 of 250 JE patients (*5*,*6*). JE transmission is a risk in 24 countries, totaling 3 billion persons at risk (*7*). JE is associated with immense social and financial burden because of the severe neuropsychiatric sequelae and the need for physical and cognitive therapy throughout the patient’s lifetime (*4*). Previous economic evaluations of JE have examined the cost of illness for acute JE and sequelae in Cambodia, China, India, Indonesia, and Nepal. However, few of those studies assessed how the cost of sequelae care changes with level of sequelae severity and over time (*8*–*11*). Only the study in Nepal explored how the economic burden of JE sequelae care differed by level of disability; however, the cost data reflected only sequelae care provided within 12 months after hospital discharge (*11*). To estimate the annual cost of sequelae care across a longer period, another study in China surveyed caregivers of JE patients with sequelae the first year after hospital discharge, but that study did not examine how those costs varied with the sequelae severity (*10*). In addition, published cost-of-illness data for acute JE and sequelae come primarily from studies conducted >1 decade ago and, given improvements during this time may not reflect the current standard of care in hospitals, rehabilitation facilities, and other settings.

The first JE outbreak in Bangladesh was reported from the central part of the country in 1977 (*12*). Not until 2003–2005 was a systematic hospital-based study conducted in 4 hospitals to assess the JE etiologies (*13*). That study reported that 55% of JE patients were from the northwestern part of the country and that most patients resided in rural areas (*13*). Among all patients, 65% reported either physical or cognitive disability/sequelae 4–6 weeks after hospital discharge (*13*). In Bangladesh, JE surveillance was conducted as part of hospital-based acute encephalitis surveillance in tertiary care hospitals during 2007–2016 and identified 548 (8%) JE patients among 6,525 tested patients (*14*). The 548 patients resided in 36 of the 64 Bangladesh districts (*14*). Most JE cases in Bangladesh occurred annually, during July–November (*14*). The median age of JE patients was 30 years and 35% were <15 years of age (*14*). There is no specific treatment for JE; care is largely supportive to relieve signs/symptoms (*2*). With this study, we measured the cost of acute JE care and initial and long-term rehabilitation and sequelae care according to severity, from the perspective of the household and the healthcare system, and assessed the effects of JE-related expenses on household financial health.

The data collection team obtained written informed consent from all participants or their guardians (for children) included in the study. The Ethical Review Committee of icddr,b and the Western Institutional Review Board and Copernicus group (WCG IRB) approved the study protocol.

## Materials and Methods

### Study Sites and Population

During October 2021–April 2022, we recruited patients who had had laboratory-confirmed JE during the previous 10 years from the JE surveillance of Maternal, Newborn, Child and Adolescent Health and the Institute of Epidemiology, Diseases Control and Research of the Government of Bangladesh (Figure). The patients had received treatment for the JE episode at public hospitals. Each JE patient was categorized into 1 of 3 groups: acute JE, initial sequelae, and long-term sequelae (Table 1), hereafter referred to as acute patients, initial sequelae patients, and long-term sequelae patients. We randomly selected patients in each group until the target sample size was reached. Acute patients were those discharged during November 2020–December 2021, initial sequelae patients those discharged November 2019–December 2021, and long-term sequelae patients those discharged November 2011–October 2019.

**Figure:**
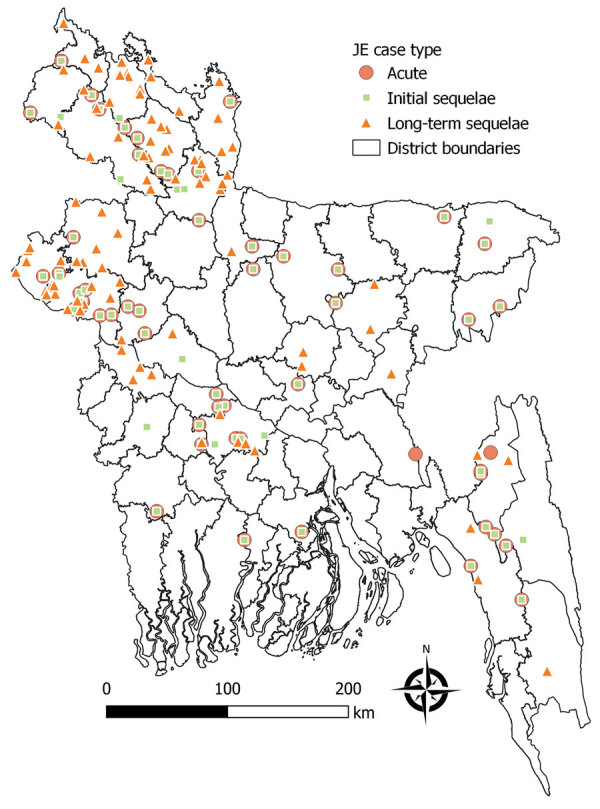
Locations of patients enrolled in study of costs of acute and sequelae care for Japanese encephalitis (JE) patients, Bangladesh, 2011–2021.

**Table 1:** Time frame considered for caregivers for patients with acute onset, initial sequelae, and long-term sequelae of Japanese encephalitis, Bangladesh, 2011–2021

Time frame	Acute (onset)	Initial sequelae (first 2 y after discharge)	Long-term sequelae (2–10 y after discharge)
Time elapsed at time of enrollment	Up to 12 mo since hospital discharge (with a preference for more recent cases)	3–24 mo since hospital discharge	25–120 mo since hospital discharge
Retrospective reference period or event for data collection	Prehospitalization costs to trip home from hospital and follow-up visits up to 90 d after discharge	90 d (3 mo) before enrollment	90 d (3 mo) before enrollment

### Sample Size

We used the standard sample size formula for estimating a population mean (*15*) to calculate the target sample size (Appendix). By setting precision at ±10% of the mean cost, setting coefficient of variation of 0.5, and considering the estimated 195 annual JE patients/year since 2011 and the Bangladesh case-fatality rate of 10% (*13*,*16*), we estimated a total sample size of 229: 64 patients for the acute group, 75 patients for the initial sequelae group, and 90 patients for the long-term sequelae group. Because of the small number of JE patients identified during the COVID-19 pandemic (November 2019–December 2021), to achieve the target sample size we administered acute and initial sequelae questionnaires to enrolled participants.

### Sequelae Assessment

The team used the Liverpool Outcome Score (LOS) (*17*), a tool to measure outcome after encephalitis, to screen for JE sequelae. The LOS had a total of 15 questions divided into 2 sections. During the first section, administered by telephone, we asked the parent/caregiver 10 questions, after which we recruited patients experiencing some level of sequelae to participate in the study, as indicated by an outcome score of <5 in the first LOS section. During the second section, to obtain total LOS outcome score we visited the households to observe the patient performing simple motor tasks. For acute patients, we assessed severity of illness by their level of consciousness. We categorized patient condition as conscious (mild illness), altered consciousness (moderate illness), or unconscious (severe illness).

### Data Collection Procedure

We collected primary and secondary data. During primary data collection, we interviewed consenting caregivers or patients with regard to socioeconomic status, costs of care, strategies for responding to JE-related expenses, and effect on the family. During secondary data collection, from consenting participants and with hospital approval, we extracted direct medical costs from the patient files, main order book, and register books of the hospitals where the patients received care.

### Cost Estimates

We measured direct medical, nonmedical, and indirect costs. Direct medical costs included medicines (prescription and over-the-counter drugs, homeopathics, traditional medicines), diagnostics, procedures, and interventions (in hospital and from other providers such as acupuncturists and physical therapists), facility/provider fees (registration, consultation, hospital bed), and other informal payment. For direct medical costs, we included informal payments because most informal payments were made for various direct medical services (e.g., payments to nurses and cleaning and ward support staff for help with availing a wheelchair or a bed at the hospital). Direct nonmedical costs included travel, lodging, meals, and other miscellaneous costs. Indirect costs were measured by household income lost while providing care for patients and accompanying them to healthcare facilities. We collected information for each day of income lost by patients and their household members while seeking healthcare facilities and providers and while caring for the patients. We estimated the household income lost by multiplying the number of total working days missed by the patient and the caregivers because of the disease and the income that they had to forgo each day. We also collected information about missed school days by patients and other household members; we did not monetize that cost but rather reported it as effect on the family’s overall well-being. For acute patients, we collected the cost of prehospitalization, hospitalization, discharge, and follow-up visits up to 90 days after discharge. For sequelae patients, we collected costs for JE-related care in the previous 90 days.

We considered the healthcare system perspective and the household perspective together representing a societal perspective. Healthcare system costs were direct medical costs covered by the healthcare system. Costs from the household perspective included direct medical costs and direct nonmedical costs that were paid out-of-pocket by the patients and their families. Indirect costs were also borne by the households. The societal costs included all the aforementioned costs and were paid by the household along with the government healthcare system.

### Data Analyses

We used descriptive statistics to analyze costs by patient age group, patient sex, patient wealth quintile, and illness severity. We calculated the costs by multiplying the quantity of the items by unit costs, which were charges from providers or payment made by the families. Costs for patients in the acute group were the average total costs for the acute episode, and costs for those in the initial sequelae and long-term sequelae groups were average monthly costs. We calculated the proportion of expenditure for JE sequelae to household income by dividing monthly costs for sequelae by household monthly income, multiplied by 100 for a patient. All costs were collected in Bangladesh Taka (BDT) and converted to US dollars. The conversion rate was adjusted to reference year 2021, and the exchange rate was 85.08 BDT to 1 US dollar (*18*). To assess the group differences with a 5% level of significance (i.e., p<0.05), we performed the Mann-Whitney U-test for binary group variables and the Kruskal-Wallis test for polytomous group variables. 

## Results

We collected data for 3 study groups from a total of 157 patients. The acute care group consisted of 55 patients, initial sequelae 65 patients, and long-term sequelae 90 patients; 53 patients were considered for acute and initial care costs and received both the acute and initial sequelae questionnaires. The average duration of initial sequelae care was 9.24 (median 6.41) months and for long-term sequelae care was 62.29 (median 64.17) months.

Most patients were <18 years of age (Table 2). More patients were male than female. Average monthly income was US $171–$193 and for more than half the families in all groups was US $118–$353 (Table 2).

**Table 2:** Sociodemographic characteristics of patients with acute onset, initial sequelae, and long-term sequelae of Japanese encephalitis, Bangladesh, November 2011–December 2021

Characteristic	Acute, n = 55	Initial sequelae, n = 65	Long-term sequelae, n = 90
Patient			
Age, y, mean (SE)	20 (3)	22 (3)	30 (3)
Age group, y			
<18	34 (62)	38 (58)	40 (44)
18–30	7 (13)	8 (12)	11 (12)
31–40	1 (2)	2 (3)	7 (8)
41–50	4 (7)	4 (6)	6 (7)
51–60	4 (7)	7 (11)	12 (13)
>60	5 (9)	6 (9)	14 (16)
Sex			
M	29 (53)	35 (54)	5 (62)
F	26 (47)	30 (46)	34 (38)
Household			
Location			
Rural	51 (93)	59 (91)	81 (90)
Urban	4 (7)	6 (9)	4 (4)
Peri-urban	NA	NA	5 (6)
No. members, mean (SE)	5 (0)	5 (0)	5 (0)
No. income earners, mean (SE)	1 (0)	1 (0)	1 (0)
Monthly income, mean (SE)	187 (21)	193 (20)	171 (11)
Income groups			
<$117.54	20 (36)	22 (34)	39 (43)
$117.55–352.1	31 (56)	38 (58)	46 (51)
$352.62–587.68	3 (5)	3 (5)	5 (6)
>$587.68	1 (2)	2 (3)	0
Monthly expense, mean (SE)	203 (11)	233 (18)	202 (9)
Monthly expense, mean (SE)†			
<$117.54	6 (11)	9 (14)	15 (17)
$117.55–235.07	31 (56)	30 (46)	45 (50)
$235.08–352.61	15 (27)	16 (25)	25 (28)
>$352.62	3 (5)	10 (15)	5 (6)
Monthly healthcare expense, mean (SE)	28 (3)	34 (6)	27 (2)
Caregiver respondent			
Relationship	51 (93)	61 (94)	78 (87)
Mother	19 (35)	22 (34)	26 (29)
Father	18 (33)	19 (29)	29 (32)
Legal guardian	8 (15)	13 (20)	10 (11)
Grandparent	1 (2)	1 (2)	4 (4)
Aunt/uncle	4 (7)	4 (6)	4 (4)
Sibling	1 (2)	2 (3)	5 (6)
Sex			
M	30 (55)	33 (51)	59 (66)
F	25 (45)	32 (49)	31 (34)
Age, y, mean (SE)	38 (2)	37 (1)	40 (1)
Age group, y			
18–30	16 (29)	20 (31)	18 (20)
31–40	21 (38)	25 (38)	34 (38)
41–50y	12 (22)	12 (18)	24 (27)
51–60	4 (7)	7 (11)	8 (9)
>60	2 (4)	1 (2)	6 (7)
Education			
None	12 (22)	15 (23)	23 (26)
Primary school	9 (16)	14 (22)	23 (26)
Secondary school	24 (44)	18 (28)	23 (26)
Higher secondary school	8 (15)	15 (23)	11 (12)
Some college or technical school training	0	0	1 (1)
Bachelor’s degree or higher	2 (4)	3 (5)	9 (10)

*Values are no. (%) patients except as indicated. All monetary amounts are in US dollars. NA, not applicable (other groups located in urban and rural areas only.) †Odd US$ are used in income categories because they were based on Bangladesh currency.

### Societal Costs 

The average societal cost of an acute JE episode was US $929 (median [SE] US $909 [$68]) (Table 3). The percentage of total costs for patients with acute JE was 40% (US $370) for direct medical costs and 38% (US $351) for indirect costs. For initial sequelae patients, the average monthly total cost was US $75 (median [SE] US $33 [$13]), and for long-term sequelae patients, US $47 (median [SE] US $14 [$8]). For initial sequelae and long-term sequelae patients, indirect cost was the highest and was incurred for 60% (39/65) of the initial sequelae patients and 53% (48/90) of the long-term sequelae patients (Table 3). Drug and diagnostic costs were the most common—and the highest direct medical cost—for acute patients. However, drug and consultation/registration fees were low but most common for initial and long-term sequelae care. Costs for procedures/intervention (physical therapy) were highest for the initial sequelae group but were incurred for only 6% (4/65) of the initial sequelae patients. Medical equipment (braces, wheelchairs, sticks) was most costly for long-term sequelae patients, and costs were incurred for 11% (10/90) of those patients (Table 3). A total of 5% of the initial sequelae patients and 7% of the long-term sequelae patients reported that they stopped seeking care for issues associated with JE illness and consequences.

**Table 3:** Societal and household costs of acute, initial, and long-term sequelae Japanese encephalitis patients, Bangladesh, November 2011–December 2021*

Cost parameter	Acute, n = 55					Initial sequelae, cost/mo, n = 65					Long-term sequelae, cost/mo, n = 90			
	No.	Mean (SE)		Median		No.	Mean (SE)		Median		No.	Mean (SE)	Median	
Cost to society														
Direct medical	55	370 (32)		298		62	18 (3)		9		80	10 (2)	3	
Drugs	55	219 (25)		167		62	8 (1)		4		79	7 (1)	1	
Diagnostic tests	53	116 (12)		89		12	15 (5)		8		7	5 (2)	2	
Procedures/interventions	18	22 (9)		6		4	25 (12)		20		3	8 (8)	1	
Consult/registration fees	48	11 (2)		7		25	9 (2)		4		19	4 (2)	2	
Medical equipment	8	14 (4)		9		10	10 (3)		7		10	11 (4)	6	
Other medical	11	3 (1)		2		4	7 (4)		3		4	2 (0)	2	
Direct nonmedical	55	221 (20)		180		58	11 (2)		6		72	8 (2)	3	
Travel	55	97 (10)		82		55	4 (1)		2		56	2 (0)	1	
Lodging and meals	52	111 (13)		71		32	8 (2)		2		34	6 (2)	3	
Telephone	54	10 (1)		8		24	0 (0)		0		30	0 (0)	0	
Patient care	13	30 (9)		12		14	8 (2)		4		15	11 (3)	6	
Housework	2	3 (3)		3		6	2 (0)		2		9	4 (2)	2	
Other care/tasks	2	47 (41)		47		3	3 (1)		2		6	6 (2)	3	
Indirect	53	351 (48)		250		39	75 (17)		29		48	54 (11)	18	
Total cost to society	55	929 (68)		909		62	75 (13)		33		84	47 (8)	14	
Cost to household														
Direct medical	54	270 (24)		224		61	17 (2)		9		79	10 (2)		2
Drug	54	136 (18)		100		61	7 (1)		4		77	7 (1)		2
Diagnostic tests	51	107 (11)		89		10	17 (5)		11		7	4 (2)		2
Procedures/interventions	12	22 (12)		5		4	25 (12)		2		3	8 (8)		1
Consult/registration fees	47	10 (2)		7		23	8 (2)		5		19	4 (2)		2
Medical equipment	8	15 (4)		9		10	10 (3)		7		10	11 (4)		6
Other medical	11	3 (1)		2		4	7 (4)		3		4	2 (0)		2
Direct nonmedical	55	221 (20)		180		58	11 (2)		6		72	8 (2)		3
Travel	55	97 (10)		82		55	4 (1)		2		56	2 (0)		1
Lodging and meals	52	111 (13)		71		32	8 (2)		2		34	6 (2)		3
Telephone	54	10 (1)		8		24	0 (0)		0		30	0 (0)		0
Patient care	13	30 (9)		12		14	8 (2)		4		15	11 (3)		6
Housework	2	3 (3)		3		6	2 (0)		2		9	4 (2)		2
Other care/tasks	2	47 (41)		74		3	3 (1)		2		6	6 (2)		3
Indirect costs	53	351 (48)		250		39	75 (17)		29		48	54 (11)		18
Total cost to household	55	825 (64)		797		62	74 (13)		33		84	47 (8)		14
Cost to healthcare system														
Drugs	54	88 (14)		52		11	4 (20)		1		6	5 (3)		2
Diagnostic tests	18	39 (10)		18		2	2(1)		2		1	4 (0)		4
Procedures/interventions	11	13 (4)		5		NA	NA		NA		NA	NA		NA
Consult/registration fee	5	11 (4)		8		3	11 (8)		4		NA	NA		NA
Total cost to healthcare system	54	106 (14)		66		13	6 (3)		3		7	5 (2)		3

*Costs are in US$. Acute group costs are the total amount, and sequelae group costs are monthly amounts. The cost values are calculated for persons for whom such costs were incurred, indicated by the sample size n in the table. NA, not applicable.

When study participants were analyzed by age, sex, illness severity, and wealth, the cost of JE was significantly higher (p = 0.011) among male than female patients (Table 4). For acute JE care, the cost increased with severity levels, although not significantly (p = 0.064). For initial and long-term JE sequelae care, the cost for patients with mild illness was significantly less than for those with moderate and severe sequelae (p = 0.038 for initial group and p = 0.035 for long-term group). The costs did not significantly differ among age groups and wealth categories, except for the long-term sequelae among those 41–50 years of age, which may be affected by the small number of cases (n = 5).

**Table 4:** Cost of illness by patient age, sex, illness severity, and wealth index of Japanese encephalitis patients with acute onset, initial sequelae, and long-term sequelae, Bangladesh, November 2011–December 2021*

Categories*	Acute, n = 55				Initial sequelae, n = 65				Long-term sequelae, n = 90		
	No.	Mean (SE)	Median		No.	Mean (SE)	Median		No.	Mean (SE)	Median
Patient age, y											
<18	34	898 (90)	883		35	60 (13)	23		36	19 (5)	5
18–30	7	1,177 (231)	1,318		8	54 (20)	34		10	45 (26)	13
31–40	1	1,157	1,157		2	335 (271)	335		7	97 (32)	64
41–50	4	876 (263)	965		4	91 (35)	90		5	148 (62)	194
51–60	4	871 (186)	911		7	128 (45)	75		12	64 (23)	41
>60	5	835 (166)	968		6	31 (12)	23		14	47 (14)	23
p value	0.810				0.123				0.002		
Patient sex											
M	30	1,089 (100)	1,076		32	91 (22)	43		55	55 (11)	24
F	25	738 (74)	790		30	59 (14)	33		29	32 (9)	9
p value	0.011				0.35				0.069		
Illness severity											
Mild	20	720 (74)	747		11	27 (11)	11		11	9 (3)	8
Moderate	11	937 (96)	1,023		16	57 (13)	31		37	60 (14)	17
Severe	24	1,100 (127)	1,006		35	99 (22)	64		36	46 (10)	29
p value	0.064				0.038				0.035		
Wealth quantile											
Poor	11	865 (147)	837		12	84 (18)	85		16	67 (23)	12
Lower middle	12	924 (93)	953		12	90 (90)	22		16	33 (9)	17
Middle	10	982 (242)	709		13	37 (12)	23		18	56 (20)	13
Upper middle	11	1,076 (146)	963		12	75 (20)	74		17	20 (6)	12
Rich	11	804 (135)	968		13	91 (44)	40		17	61 (20)	35
p value	0.742		0.403						0.518		

*Costs are in US$. Acute group costs are the total amount, and sequelae group costs are monthly amounts.

### Household Costs 

The average household cost of an acute JE episode was US $825 (median [SE] US $797 [$64]), which accounts for 89% (825/929) of the total cost of illness (Table 3). For average household costs, the incurred indirect expense was 43% (US $351), and direct medical cost was 33% (US $270) of the total cost of acute JE. Nearly 100% of the costs of sequelae care were borne by the families; costs were US $74 (median [SE] US $33 [$13]) for initial and US $47 (median [SE] US $14 [$8]) for long-term sequelae patients (Table 3). The average monthly cost for initial sequalae consisted of 56% of the household monthly income. For long-term sequalae care, the monthly cost consisted of 44% of the household monthly income.

### Healthcare System Costs 

The average health system cost of an acute JE episode was US $106 (median [SE] $66 [$14]), which accounts for 11% (106/929) of the total societal cost (Table 3). The average healthcare system costs of sequelae care were US $6 per month (median [SE] US $3 [$3]) for the initial sequelae group and US $5 per month (median [SE] US $3 [$2]) for the long-term sequelae group (Table 3).

### Coping Strategies 

Most families reported that they coped with the expenses of the illness by using savings and then borrowing money (Table 5). For expenses during the acute phase, families that used savings spent an average (SE) of US $403 ($75). Those who sought a loan required an average (SE) of US $734 ($112), not accounting for interest. Those who sold household assets used an average (SE) of US $683 ($146) to cover expenses. The economic burden was experienced not only during the acute stage but also during the sequelae stages because most families used savings and one third took a loan for monthly JE-related expenses. To cover expenses, the families also received donations from relatives, other household members, friends, charity, and local community or government leaders.

**Table 5:** Coping strategies for the expenses of acute care, initial sequelae, and long-term sequelae for JE patients, Bangladesh, November 2011–December 2021*

Coping strategy	Acute care			Initial sequelae			Long-term sequelae	
	No. (%)	Mean, total (SE)		No. (%)	Mean, mo (SE)		No. (%)	Mean, mo (SE)
Source of funds for JE expenses								
Using savings	49 (89)	403 (75)		59 (91)	41 (7)		71 (79)	47 (15)
Borrowing	40 (73)	734 (112)		14 (22)	195 (52)		26 (29)	385 (136)
Donations	28 (51)	261 (72)		14 (22)	28 (8)		14 (16)	22 (7)
Selling assets	26 (47)	683 (146)		4 (6)	205 (64)		10 (11)	885 (253)
Cutting down expenses	3 (5)	129 (58)		7 (11)	73 (31)		16 (18)	23 (8)
Sustained debt								
From acute period	NA	NA		53 (82)	NA		82(91	NA
Still paying	NA	NA		22 (34)	96 (33)		34 (38)	74 (13)

*Costs are in US$. JE, Japanese encephalitis; NA, not applicable.

Most (82%, 53/65) respondents reported that they had sustained debt from borrowing during the acute phase of the illness (Table 5). The average monthly payment for sustained debt was US $96 for the initial sequelae group and US $74 for the long-term group. Borrowing from relatives was most common during the acute phase, and a bank was the common source during the sequelae phase (Table 3). Families most commonly identified their plan to repay debt as working extra hours.

### Effects on the Family’s Overall Well-Being

Most families of acute and sequelae patients reported that the effects were extreme, particularly for expenses as well as stress and fear associated with the disease (Table 6). An extreme effect on missed school was reported by 49% of the acute, 43% of the initial, and 42% of the long-term sequelae patients (Table 6). Altogether, the patients and caregivers of acute patients missed 92 school days during the entire episode. On the other hand, 26% of the initial patients (19 days/mo) and 19% of the long-term patients (21 days/mo) missed school days (Table 6).

**Table 6:** Effects of on acute care, initial sequelae, and long-term sequelae of Japanese encephalitis patients on family’s overall well-being, Bangladesh, November 2011–December 2021

Level of effect	Acute, no. (%)	Initial sequelae, no. (%)	Long-term sequelae, no. (%)
Expenses associated with the disease			
None	1 (2)	1 (2)	0
Little	3 (5)	4 (6)	2 (2)
Moderate	7 (13)	6 (9)	10 (11)
Extreme	44 (80)	54 (83)	78 (87)
Income of members in the household	3 (5)	5 (8)	4 (4)
Little	4 (7)	4 (6)	4 (4)
Moderate	8 (15)	13 (20)	12 (13)
Extreme	40 (73)	43 (66)	70 (78)
Missed school because of to illness*			
Patient’s missed school (d missed)	20 (80)	16 (20)	15 (22)
caregivers' missed school (d missed)	6 (116)	1 (1)	4 (10)
Total missed school, no. (d missed)	25 (92)	17 (19)	17 (21)
None	25 (45)	32 (49)	38 (42)
Little	1 (2)	1 (2)	6 (7)
Moderate	2 (4)	4 (6)	8 (9)
Extreme	27 (49)	28 (43)	38 (42)
Stress and fear associated with the disease			
None	1 (2)	0	3 (3)
Little	1 (2)	2 (3)	5 (6)
Moderate	5 (9)	8 (12)	11 (12)
Extreme	48 (87)	55 (85)	71 (79)

*Values are no. (%) patients except as indicated. Missed school days were reported as total for acute care patients and monthly for sequelae patients.

## Discussion

We provide an estimate of the economic burden of JE in Bangladesh. The average societal cost of an acute JE episode estimated from 55 patients was US $929 (median US $909), and the average household cost was US $825 (median US $797). The average monthly societal cost of initial sequelae care, estimated from 65 patients, was US $75 (median US $33), and average monthly household cost was US $74 (median US $33). The average monthly household cost of long-term sequelae care, estimated from 90 patients, was US $47 (median US $14). Although during the acute phase, approximately one tenth of the cost was borne by the healthcare system, almost all costs during the sequelae phase were borne by the households. The monthly cost for sequelae care was measured over 3 months and then assumed to remain constant over the rest of the patient’s life. The monthly costs were then aggregated over the long term and created substantial economic burden for the families.

Similar to findings of a study conducted in India (*19*), our study also noted that cost per case for sequelae patients decreases over time. In addition, similar to findings of another study (*20*), we found that among acute patients, direct costs were higher than indirect costs, which might be explained by direct medical costs consisting of medication, diagnoses, and other procedures. On the other hand, similar to findings of another study in China (*10*), we found that among sequelae patients, indirect costs were higher than direct medical costs, which might result from more wage loss because the patient and the caregiver were unable to be involved in income-generating activities.

More than half of the JE patients in Bangladesh were children (*2*,*14*). The cost of a child disabled for life by JE is a heavy burden for the families. For a patient who recovers completely, the treatment is expensive in terms of financial as well as productivity loss of the child’s caregiver. Similar to findings of other studies (*20*), our study found a higher cost for male than female patients. Those findings were consistent within the context of Bangladesh because discrimination against women exists for health-associated behavior (*21*). In Bangladesh, male household members are usually prioritized for healthcare-seeking practices (*21*,*22*). In low-income countries, a household usually spends 2%–5% of its income on healthcare (*23*). In our study, most of the cost was borne by the households for acute (89%), initial (99%), and long-term (100%) sequelae care, which is similar to findings from a separate study (*24*). Furthermore, for patients with JE sequelae, an average of 44%–56% of the household monthly income was spent on healthcare, indicating a catastrophic expenditure for the families. In 2017, the illness cost borne by households (out-of-pocket expenditure) was 74% of the total health expenditure in Bangladesh (*25*). During the same year, 3.3% of the population of Bangladesh was pushed into poverty because of out-of-pocket health expenditures (*26*). Previous studies have shown that most of the cost of healthcare in Bangladesh is paid by families (*27*,*28*), despite access to government healthcare, which is purportedly free of charge yet requires payment of unofficial fees (*27*). When patient treatment depends on the ability to pay, patients living in low-income families face devastating consequences. We also noted that some sequelae patients stopped seeking treatment for JE. The level of expenditure of JE, from acute phase to prolonged treatment, often forces households to use savings, sell assets (e.g., livestock), work extra hours, borrow money, and continue to pay high debts, thus leading to impoverishment (*29*). That downward spiral of assets and income loss may lead some families into abject poverty and subsequently perpetuate the cycles of generational poverty (*30*). Moreover, the extreme effects on psychosocial health may also cause different illnesses among family members and may affect the health and well-being of the overall family.

Among our study limitations, recall bias was a concern for collecting cost data from participants by using a micro-costing approach (most precise assessment). We tried to limit recall bias in several ways (e.g., extracting direct medical costs from hospital records rather than asking participants, reporting cost/visit rather than cost/episode). In addition, to reduce recall bias, we used a 90-day retrospective reference period for the initial and long-term sequelae costs, as recommended in the literature (*31*). Out of respect for the families, we did not include cases that ended in death; thus, cost of death is out of the scope of our study. Although not being able to find the required number of patients for acute- and initial-phase cost data collection limited our generalizability, we enrolled all the available JE patients for those 2 phases. 

Our findings are useful for providing information about the magnitude of economic burden and the effects of JE among the affected families in Bangladesh. In addition, our findings can be used with regard to JE prevention, which plays a crucial role in alleviating the burden of the disease. The most effective and sustainable way to prevent JE in humans is vaccination. Updated evidence on the cost of JE illness, and on initial and long-term rehabilitation and sequelae care by severity, will further underscore the value of JE vaccination and can be used for national decision-making for JE vaccine introduction and sustainability. Our findings that the average societal cost of an acute JE episode was US $929, average monthly cost of initial sequelae was US $75, and average monthly cost of long-term sequelae was US $47 provide an economic estimation that can be used for policy decisions with regard to vaccine introduction in Bangladesh. 

Appendix Additional methods and results for study of costs of acute and sequelae care for Japanese encephalitis patients, Bangladesh, 2011–2021.
