# Supplementary material for: Cost of Acute and Sequelae Care for Japanese Encephalitis Patients, Bangladesh, 2011–2021
Source: Emerg Infect Dis. 2023 Dec;29(12):2488–97. doi: 10.3201/eid2912.230594 (PMC10683813; doi:10.3201/eid2912.230594)
Supplement: Appendix — Additional methods and results for study of costs of acute and sequelae care for Japanese encephalitis patients, Bangladesh, 2011–2021. [file 23-0594-Techapp-s1.pdf]

Article DOI: https://doi.org/10.3201/eid2912.230594

EID cannot ensure accessibility for supplementary materials supplied by authors. Readers who have difficulty accessing supplementary content should contact the authors for assistance.

Cost of Acute and Sequelae Care for Japanese Encephalitis Patients, Bangladesh, 2011–2021

Appendix

Sample size formula

We use the standard sample size formula for estimating a population mean (*1*) to calculate the target sample size:

$$n = ceiling\left[\left(\frac{precision^2}{CV^2 \times Z_{1-\alpha/2}^2} + \frac{1}{N_0}\right)^{-1}\right]$$

Setting precision at +/− 10% of the mean cost, coefficient of variation of 0.5 and considering the estimated 195 annual JE patients per year since 2011, and Bangladesh case fatality rate of 10% (*2,3*), we estimated total sample size of 229 with 64 patients for acute group, 75 patients for initial sequelae group, and 90 patients for long-term sequelae group. Due to the small number of JE patients identified during the COVID-19 pandemic (between November 2019 and December 2021), we administered both acute and initial sequelae questionnaires to enrolled participants to achieve the target sample size.

References

1. Thompson SK. Sampling. 3rd ed. Hoboken, New Jersey: John Wiley & Sons; 2012.

2. Hossain MJ, Gurley ES, Montgomery S, Petersen L, Sejvar J, Fischer M, et al. Hospital-based surveillance for Japanese encephalitis at four sites in Bangladesh, 2003-2005. Am J Trop Med Hyg. 2010;82:344–9. PubMed https://doi.org/10.4269/ajtmh.2010.09-0125

3. World Health Organization. Japanese encephalitis - Reported cases by country. Global Health Observatory data repository. 2020 [cited 2022 Oct 5]. https://apps.who.int/gho/data/node.main-searo.WHS3_42?lang=en

Appendix Table 1. Societal and household costs (BDT) of the acute, initial and long-term sequelae Japanese encephalitis cases of Bangladesh from November 2011 through December 2021

Cost parameter*	Acute (total), N = 55			Initial sequelae (monthly), N = 65			Long-term sequelae (monthly), N = 90		
Societal cost		Mean (SE)	Median		Mean (SE)	Median		Mean (SE)	Median
Direct medical cost	55	31,442 (2,755)	25,387	62	1,520 (217)	784	80	864 (142)	250
Drug	55	18,643 (2,154)	14,192	62	674 (108)	381	79	609 (109)	120
Diagnostic test	53	9,886 (997)	7,600	12	1,263 (392)	708	7	384 (180)	167
Procedure/intervention	18	1,914 (760)	500	4	2,168 (1016)	1,668	3	706 (647)	67
Consult/registration fee	48	909 (182)	618	25	733 (189)	333	19	332 (145)	133
Medical equipment	8	1,225 (373)	800	10	809 (279)	633	10	916 (382)	492
Other medical costs	11	232 (65)	200	4	554 (379)	250	4	173 (32)	147
Direct non-medical cost	55	18,826 (1,679)	15,290	58	914 (160)	502	72	646 (133)	217
Travel	55	8,293 (829)	6,970	55	354 (71)	150	56	146 (33)	57
Lodging and meals	52	9,426 (1,110)	6,050	32	686 (206)	200	34	502 (131)	225
Telephone	54	877 (110)	710	24	26 (6)	17	30	24 (4)	18
Care of patient	13	2,565 (770)	1,000	14	657 (204)	333	15	942 (251)	500
Doing housework	2	235 (215)	235	6	172 (22)	175	9	363 (147)	200
Other care/tasks	2	3,975 (3,525)	3,975	3	244 (44)	200	6	522 (202)	292
Indirect costs	53	29,875 (4,116)	21,300	39	6,381 (1,469)	2,500	48	4,629 (905)	1,533
Cost of illness of society	55	79,055 (5,761)	77,346	62	6,389 (1,127)	2,837	84	4,022 (657)	1,179
Household cost									
Direct medical cost	54	23,008 (2,026)	19,094	61	1,428 (209)	756	79	837 (141)	200
Drug	54	11,536 (1,495)	8,537	61	621 (107)	323	77	591 (108)	131
Diagnostic test	51	9,091 (957)	7,600	10	1,484 (438)	975	7	336 (174)	167
Procedure/intervention	12	1,893 (1,048)	407	4	2,167 (1,016)	168	3	706 (647)	67
Consult/registration fee	47	832 (178)	615	23	672 (154)	400	19	332 (145)	133
Medical equipment	8	1,225 (373)	800	10	809 (279)	633	10	916 (382)	492
Other medical costs	11	232 (65)	200	4	554 (379)	250	4	173 (32)	147
Direct non-medical cost	55	18,826 (1,679)	15,290	58	914 (160)	502	72	646 (133)	217
Travel	55	8,293 (829)	6,970	55	354 (71)	150	56	146 (33)	57
Lodging and meals	52	9,426 (1,110)	6,050	32	686 (206)	200	34	502 (131)	225
Telephone	54	877 (110)	710	24	26 (6)	17	30	24 (4)	18
Care of patient	13	2,565 (770)	1,000	14	657 (204)	333	15	942 (251)	500
Doing housework	2	235 (215)	235	6	172 (22)	175	9	363 (147)	200
Other care/tasks	2	3,975 (3,525)	3,975	3	244 (44)	200	6	522 (202)	292
Indirect costs	53	29,875 (4,116)	21,300	39	6,381 (1,469)	2,500	48	4,629 (905)	1,533
Cost of illness of household	55	70,203 (5,483)	67,824	62	6,274 (1,128)	2,837	84	3,986 (653	1,179
Health system cost									
Drug	54	7,452 (1,170)	4,406	11	358 (183)	105	6	446 (235)	152
Diagnostic test	18	3,350 (821)	1,500	2	158 (108)	158	1	333 (0)	333
Procedure/intervention	11	1,067 (371)	400						
Consult/registration fee	5	899 (328)	699	3	951 (720)	333			
Cost of illness of health system	54	9,016 (1,231)	5,636	13	546 (218)	217	7	430 (199)	239

*Acute group is total amount and sequelae groups is monthly amount.

Appendix Table 2. Societal cost of illness (BDT) by age, sex, severity and wealth index of the acute, initial and long-term sequelae Japanese encephalitis cases of Bangladesh from November 2011 through December 2021

	Acute (total), N = 55			Initial sequelae (monthly), N = 65			Long-term sequelae (monthly), N = 90		
Categories*	No.	Mean (SE)	Median	No.	Mean (SE)	Median	No.	Mean (SE)	Median
Age									
<18 y	34	76,429 (7,623)	75,147	35	5,116 (1,137)	1,997	36	1,614 (386)	442
18–30 y	7	100,167 (19,614)	112,158	8	4,635 (1,681)	2,882	10	3,843 (2,237)	1,078
31–40 y	1	98,407	98,407	2	28,479 (23,029)	28,479	7	8,235 (2,738)	5,473
41–50 y	4	74,500 (22,348)	82,063	4	7,736 (2,958)	7,672	5	12,603 (5,316)	16,465
51–60 y	4	74,122 (15,810)	77,472	7	10,925 (3,832)	6,347	12	5,427 (1,932)	3,523
60+ years	5	71,077 (14,131)	82,327	6	2,598 (1,005)	1,921	14	3,964 (1,221)	1,962
P-value		*0.810*			*0.123*			*0.002*	
Sex									
Male	30	92,638 (8,497)	91,541	32	7,701 (1,854)	3,666	55	4,700 (908)	2,029
Female	25	62,756 (6,269)	67,176	30	4,989 (1,214)	2,794	29	2,735 (774)	778
P-value		*0.011*			*0.345*			*0.069*	
Severity									
Mild	20	61,293 (6,308)	63,561	11	2,262 (896)	936	11	764 (264)	707
Moderate	11	79,693 (8,146)	87,073	16	4,861 (1,096)	2,623	37	5,122 (1,176)	1,410
Severe	24	93,565 (10,834)	85,579	35	8,385 (1,848)	5,450	36	3,886 (888)	2,425
P-value		*0.064*			*0.038*			*0.035*	
Wealth quantile									
Poor	11	73,591 (12,470)	71,229	12	7,170 (1,514)	7,216	16	5,690 (1,997)	994
Lower middle	12	78,640 (7,921)	81,101	12	7,670 (3,413)	1,867	16	2,766 (754)	1,421
Middle	10	83,521 (20,615)	60,307	13	3,135 (1,020)	1,997	18	4,735 (1,703)	1,115
Upper middle	11	91,545 (12,404)	81,909	12	6,409 (1,708)	6,287	17	1,710 (504)	1,014
Rich	11	68,423 (11,491)	82,327	13	7,722 (3,781)	3,445	17	5,191 (1,676)	3,020
P-value		*0.742*			*0.403*			*0.518*	

*Acute group is total amount and sequelae groups is monthly amount.

Appendix Table 3: Source of funding for treatment of the acute, initial and long-term sequelae Japanese encephalitis cases of Bangladesh from November 2011 through December 2021

	Acute		Initial sequelae		Long-term sequelae	
Coping strategies	n	(%)	n	(%)	n	(%)
Sold assets for fund						
Livestock	16	(29)	3	(5)	6	(7)
Jewelry	2	(4)				
Machine/equipment	7	(13)				
Realty	5	(9)	1	(2)	6	(7)
Tree			1	(2)		
Source of donations						
Other household members	7	(13)	6	(9)	5	(6)
Relatives	19	(35)	5	(8)	8	(9)
Friends	6	(11)	3	(5)	2	(2)
Social welfare fund/charity					3	(3)
Political leader			1	(2)		
Source of borrowing						
Other household members	8	(15)	1	(2)	1	(1)
Relatives	22	(40)	4	(6)	12	(13)
Friends	12	(22)	3	(5)	1	(1)
Bank	4	(7)	8	(12)	14	(16)
Local money lender	11	(20)			4	(4)
Need to pay interest for loan	13	(24)	8	(12)	17	(19)
Plan for repaying loan						
Working extra hours	31	(56)	9	(14)	23	(26)
Selling assets	12	(22)	2	(3)	3	(3)
Borrowing money	5	(9)			6	(7)
Cutting down expenses	2	(4)	5	(8)	5	(6)
